# Impact of bleeding complications on length of stay and critical care utilization in cardiac surgery patients in England

**DOI:** 10.1186/s13019-019-0881-3

**Published:** 2019-04-02

**Authors:** Nawwar Al-Attar, Stephen Johnston, Nadine Jamous, Sameer Mistry, Ena Ghosh, Gaurav Gangoli, Walter Danker, Katherine Etter, Eric Ammann

**Affiliations:** 1 Department of Cardiac Surgery, Golden Jubilee National Hospital, University of Glasgow, Agamemnon St, Clydebank G81 4DY, Glasgow, UK; 2 Epidemiology, Medical Devices, Johnson & Johnson, New Brunswick, NJ USA; 3 Health Economics & Market Access, Johnson & Johnson Medical Ltd, Berkshire, UK; 4 Medical Affairs, Johnson & Johnson Medical Ltd, Berkshire, UK; 5 Mu Sigma, Bangalore, India; 6 Health Economics & Market Access, Ethicon, Somerville, NJ USA

**Keywords:** Cardiac Surgical procedures, Haemorrhage, Complications, Length of stay, Costs and cost Analysis

## Abstract

**Background:**

Bleeding is a significant complication in cardiac surgery and is associated with substantial morbidity and mortality. This study evaluated the impact of bleeding on length of stay (LOS) and critical care utilization in a nationwide sample of cardiac surgery patients treated at English hospitals.

**Methods:**

Retrospective, observational cohort study using linked English Hospital Episode Statistics (HES) and Clinical Practice Research Datalink (CPRD) records for a nationwide sample of patients aged ≥18 years who underwent coronary artery bypass graft (CABG), valve repair/replacement, or aortic operations from January 2010 through February 2016. The primary independent variables were in-hospital bleeding complications and reoperation for bleeding before discharge. Generalized linear models were used to quantify the adjusted mean incremental difference [MID] in post-procedure LOS and critical care days associated with bleeding complications, independent of measured baseline characteristics.

**Results:**

The study included 7774 cardiac surgery patients (3963 CABG; 2363 valve replacement/repair; 160 aortic procedures; 1288 multiple procedures, primarily CABG+valve). Mean LOS was 10.7d, including a mean of 4.2d in critical care. Incidences of in-hospital bleeding complications and reoperation for bleeding were 6.7 and 0.3%, respectively. Patients with bleeding had longer LOS (MID: 3.1d; *p* < 0.0001) and spent more days in critical care (MID: 2.4d; p < 0.0001). Reoperation for bleeding was associated with larger increases in LOS (MID = 4.0d; *p* = 0.002) and days in critical care (MID = 3.2d; *p* = 0.001).

**Conclusions:**

Among English cardiac surgery patients, in-hospital bleeding complications were associated with substantial increases in healthcare utilization. Increased use of evidence-based strategies to prevent and manage bleeding may reduce the clinical and economic burden associated with bleeding complications in cardiac surgery.

**Electronic supplementary material:**

The online version of this article (10.1186/s13019-019-0881-3) contains supplementary material, which is available to authorized users.

## Background

Bleeding is a significant complication in cardiac surgery, with 2–15% of open cardiac surgery patients experiencing major intra- or post-operative bleeding. [1–4] Bleeding complications are associated with worse clinical outcomes, including a higher risk of infection, ischaemic events attributable to hypo-perfusion (e.g., myocardial infarction, acute kidney injury), in-hospital mortality, and transfusion-related adverse events. [5–7] Additionally, bleeding complications are an important driver of blood product utilization in cardiac surgery. [1] Blood is a limited resource, and in England and other Western countries, 10–20% of the total blood supply is used in cardiac surgery patients. [8, 9]

The incidence and clinical burden of bleeding complications in the setting of cardiac surgery have been described in prior studies; [5–7, 10] however, there is limited contemporary data on the impact of bleeding on resource utilization in European countries, particularly for England. This study assessed the incidence of bleeding complications in cardiac surgery and their impact on post-procedure length of stay (LOS) and critical care utilization in a nationwide sample of patients treated at English hospitals.

## Methods

### Data source

De-identified records were obtained for a nationwide sample of English cardiac surgery patients from two linked databases: the Hospital Episode Statistics Admitted Patient Care (HES-APC) data warehouse and the Clinical Practice Research Datalink (CPRD). HES-APC contains administrative records for all inpatient stays at National Health Service (NHS) hospitals in England; the dataset includes information on patient demographics, diagnoses and procedures, and measures of utilization (LOS, days in critical care) associated with each inpatient stay. The CPRD database consists of electronic health record (EHR) data generated as part of standard clinical practice by participating United Kingdom (UK) general practitioners (GPs). CPRD includes EHR records of all diagnoses, observations, measurements, procedures, and prescriptions by the GP, as well as records of consultations and procedures that are shared with the GP by other healthcare providers. At present, approximately 4.4 million primary care patients representing 6.9% of the UK population contribute data to the CPRD. [11]

### Research approval and protection of human subjects

The HES-APC and CPRD study data were de-identified and obtained under license from the UK Medicines and Healthcare Products Regulatory Agency (MHRA). Throughout the duration of the study, the data were stored on encrypted, password-protected servers to protect patient confidentiality. The study protocol (number 17_091R) was reviewed and approved by the Independent Scientific Advisory Committee for MHRA Database Research. However, the interpretation and conclusions contained in this report are those of the authors alone.

### Study population

This was a retrospective, observational cohort study of cardiac surgery patients who underwent coronary artery bypass graft (CABG), heart valve replacement/repair, or an aortic operation (specifically, aortic aneurysm repair or aortic root replacement) between January 1, 2010, and February 29, 2016. Patients were identified from the HES-APC inpatient data using OPCS Classification of Surgical Operations and Procedures (4th revision) codes (see Additional file 1: Table S1). For each patient, we restricted to the first cardiac surgery observed during the study period and designated it the index operation. Cardiac surgery patients age ≥ 18 years were eligible for inclusion provided they were registered with a GP whose office contributed data to the CPRD for the 12 months prior to the index cardiac operation. The application of these selection criteria is shown in Table 1.

**Table 1:** Application of study inclusion criteria to identify eligible cardiac surgery patients

Criterion	Patients (*N*)
1. Patients who underwent coronary artery bypass graft (CABG), valve repair/replacement, or an aortic operation in English hospitals between January 1, 2010, and Feb 29, 2016, as identified from the linked HES-APC and CPRD databases.† Restrict to the first cardiac surgery (index) observed for each patient during the study period.	17,339
2. Age ≥ 18 years at index cardiac surgery.	17,142
3. Registered with a general practitioner (GP) contributing data to the CPRD for at least one year prior to index.	7774

**†** HES-APC = Hospital Episode Statistics Admitted Patient Care (HES-APC) data warehouse. CPRD = Clinical Practice Research Datalink (CPRD)

### Bleeding complication measures

- **Primary (narrow) definition of bleeding complications**: In-hospital bleeding complications were identified by the presence of International Classification of Diseases, Tenth Revision (ICD-10, as implemented in the UK) [12] diagnosis code T81.0 (“Haemorrhage and haematoma complicating a procedure, not elsewhere classified”) and/or an OPCS procedure code for a bleeding-related reoperation: T032 (“Reopening of chest and re-exploration of intrathoracic operation site and surgical arrest of postoperative bleeding”) or Y321 (“Re-exploration of organ and surgical arrest of postoperative bleeding, not otherwise classified”). In the HES-APC database, a single hospital stay or “spell” may be segmented into episodes of care based on changes in care setting (e.g., a transfer from surgery to recovery or intensive care). For identifying in-hospital bleeding complications, only diagnoses and procedures recorded during the index cardiac care episode or between the index cardiac procedure and discharge were considered.
- **Bleeding-related reoperation:** Bleeding-related reoperations were identified using the following algorithm. OCPS procedure codes T032 and Y321, which specifically denote reoperation for the arrest of bleeding, were taken as evidence of a bleeding-related reoperation. In addition, a broader set of reoperation codes (e.g., T033, “Reopening of chest and re-exploration of intrathoracic operation site, not elsewhere classified”) were counted as evidence of a bleeding-related reoperation in the presence of a concomitant diagnosis of T81.0 (“Haemorrhage and haematoma complicating a procedure, not elsewhere classified”).
- **Broad definition of bleeding complications (sensitivity analysis):** Because bleeding complications may not be coded consistently using the codes above, a broader definition of bleeding complications was considered as a sensitivity analysis. The broader definition of bleeding complications also included diagnosis codes for accidental cuts and lacerations during surgery and procedure codes for haematoma evacuation/aspiration.

Please see Additional file 1: Table S2 for a complete list of the codes used to define bleeding complications (primary/narrow and broad definitions) and bleeding-related reoperations. Blood product transfusions were not used to characterize the incidence or severity of bleeding complications in this study. Prior research has shown that blood product transfusions are not well captured in the HES-APC database. [13]

### Covariates

HES-APC and CPRD records from the index hospitalization and 12 months prior were used to measure patient demographics (age, gender, geographic region), baseline clinical characteristics, and procedure characteristics. Clinical covariates of interest included:

- Index cardiac procedure characteristics (type of surgery: CABG, valve replacement/repair, aortic procedure, or multiple procedures; elective admission status; calendar year),
- Use of oral anticoagulants, antiplatelet medications, and other drugs with meaningful effects on bleeding risk (non-steroidal anti-inflammatory drugs [NSAIDs], selective serotonin re-uptake inhibitors [SSRIs], serotonin-norepinephrine reuptake inhibitors [SNRIs]),
- Anemia and the following Charlson Comorbidity Index components: myocardial infarction, congestive heart failure, cerebrovascular disease, peripheral vascular disease, diabetes, chronic pulmonary disease, chronic renal disease, anemia, liver disease, peptic ulcer disease, connective tissue or rheumatic disease, cancer, dementia, paraplegia or hemiplegia, and HIV/AIDS. [14] For identifying patients with a history of myocardial infarction and cerebrovascular disease, only records from the 12 months prior to the index admission were used since these diagnosis codes may reflect acute in-hospital complications.
- Measures of healthcare utilization and overall health status (count of distinct prescription medications taken, count of primary care consultations, and whether the patient was hospitalized during the 12 months prior to index).

### Healthcare utilization outcomes

The primary study outcomes were post-procedure LOS and days in critical care during the index hospital stay. Post-procedure LOS (hereafter referred to simply as LOS) was defined as the time interval in days between the index cardiac procedure and hospital discharge. The critical care measure reflects the number of days (if any) that the patient spent in an intensive care unit (ICU) or high dependency unit (HDU) hospital ward. Patients in critical care require constant support and monitoring to maintain function of one or more organs. [15] As with LOS, days in critical care were measured from the date of the index cardiac procedure through discharge.

### In-hospital mortality

Because in-hospital mortality represents a competing risk that may affect the associations between bleeding complications and LOS and critical care utilization, we also evaluated the incidence of in-hospital mortality by bleeding status. In-hospital mortality was not a pre-specified endpoint in our study protocol, and we therefore consider it an exploratory outcome.

### Statistical analyses

Generalized linear models (log link; negative binomial distribution) were used to estimate the impact of bleeding complications on LOS and critical care utilization, adjusting for the patient demographic and clinical characteristics listed in the covariates section above. The impact of bleeding on LOS and critical care days was quantified using relative effect measures (adjusted multiplicative effect estimates from the generalized linear models) and absolute measures (adjusted means for patients who did vs. did not experience bleeding and the adjusted mean incremental difference [MID] calculated from model-based recycled predictions). [16] Statistical analyses were performed with SAS Enterprise Guide 7.1 for Windows (SAS Institute, Inc.; Cary, NC).

In secondary subgroup analyses, an interaction between surgery type and bleeding was added to the generalized linear models so that the impact of bleeding on LOS and critical care days could be estimated separately in patients who underwent CABG, valve replacement/repair, aortic procedures, or multiple procedures. Similarly, generalized linear models (logit link; binomial distribution) were used to quantify the association between bleeding complications and in-hospital mortality.

### Sensitivity analyses

We conducted two sensitivity analyses to examine the robustness of the study findings. First, we conducted a sensitivity analysis in which the primary analyses examining the impact of bleeding complications on LOS and critical care utilization were replicated among only patients undergoing elective admission, as those with emergency admission may not have had the opportunity to be discontinued from medications which increase the risk of bleeding, such as anticoagulants and antiplatelets. Second, we examined the impact of the primary definition of bleeding complication on LOS and critical care utilization via propensity score matching, wherein we matched patients with bleeding complications to those without bleeding complications. Specifically, we use used 1:4 variable ratio matching, via the nearest neighbor technique, with a caliper of 0.10 of the standard deviation of the propensity score. We treated all measured baseline characteristic variables as independent variables in the logistic regression used to estimate the propensity score.

## Results

### Patient characteristics

The study included 7774 eligible cardiac surgery patients from January 2010 through February 2016 (Table 1). During the index hospital episode, 3963 patients underwent CABG, 2363 valve replacement or repair, 160 aortic aneurysm repair or aortic root replacement, and 1288 had multiple cardiac procedures. Patients who underwent multiple cardiac procedures included 1029 patients with concomitant CABG and valve replacement/repair, 176 patients with valve and aortic procedures, 24 patients with CABG and an aortic procedure, and 59 patients with CABG, valve and aortic procedures. Sixty-nine percent of patients were elective admissions; 12% were emergency cases, and 19% were transfers from another hospital. Eighty-three percent of surgeries were performed with cardiopulmonary bypass.

In terms of demographics, the median patient age was 70 years; 72% were male. Twenty-three percent of patients had a history of myocardial infarction; 24% had congestive heart failure; 20% had chronic pulmonary disease; 25% had diabetes, 13% had renal disease, and 4% had anemia. Use of antiplatelet medications (60%) and oral anticoagulant drugs (13%) during the pre-index baseline period was common. Additional cohort characteristics are provided in Table 2 and Additional file 1: Table S3.

**Table 2:** Baseline patient characteristics

Covariate	All patients (*N* = 7774)	No bleeding (*N* = 7254)	Bleeding (*N* = 520)
Age in years			
•18–45	293 (4%)	278 (4%)	15 (3%)
•46–64	2187 (28%)	2063 (28%)	124 (24%)
•65 or older	5294 (68%)	4913 (68%)	381 (73%)
Gender			
•Male	5596 (72%)	5244 (72%)	352 (68%)
•Female	2178 (28%)	2010 (28%)	168 (32%)
Surgery type			
•Coronary artery bypass graft (CABG)	3963 (51%)	3762 (52%)	201 (38%)
•Valve replacement/repair	2363 (30%)	2224 (31%)	139 (27%)
•Aortic procedure	160 (2%)	136 (2%)	24 (5%)
•Multiple procedures	1288 (17%)	1132 (16%)	156 (30%)
Cardiopulmonary bypass			
•Yes	6447 (83%)	6012 (83%)	435 (84%)
•No	1327 (17%)	1242 (17%)	85 (16%)
Admission type			
•Elective	5400 (69%)	5099 (70%)	301 (58%)
•Non-elective/emergency	917 (12%)	831 (11%)	86 (17%)
•Transferred from another hospital	1457 (19%)	1324 (18%)	133 (26%)
Health conditions†			
•Myocardial infarction	1819 (23%)	1679 (23%)	140 (27%)
•Congestive heart failure	1872 (24%)	1707 (24%)	165 (32%)
•Cerebrovascular disease	456 (6%)	419 (6%)	37 (7%)
•Peripheral vascular disease	1431 (18%)	1287 (18%)	144 (28%)
•Diabetes	1951 (25%)	1842 (25%)	109 (21%)
•Chronic pulmonary disease	1559 (20%)	1463 (20%)	96 (18%)
•Renal disease	1003 (13%)	902 (12%)	101 (19%)
•Anemia	287 (4%)	265 (4%)	22 (4%)
Medication use†			
•Antiplatelet drugs	4681 (60%)	4382 (60%)	299 (58%)
•Oral anticoagulant drugs	1001 (13%)	936 (13%)	65 (13%)
•Non-steroidal anti-inflammatory drugs (NSAIDs)	448 (6%)	428 (6%)	20 (4%)
•Selective serotonin reuptake inhibitors (SSRIs)	588 (8%)	554 (8%)	34 (7%)
•Serotonin-norepinephrine reuptake inhibitors (SNRIs)	94 (1%)	89 (1%)	5 (1%)
Healthcare utilization†			
•Hospitalization	2968 (38%)	2725 (38%)	243 (47%)
• ≥ 11 primary care encounters	3779 (49%)	3548 (49%)	231 (44%)
• ≥ 6 distinct prescription medications	2319 (30%)	2157 (30%)	162 (31%)

† Health conditions, medication use, and healthcare utilization were assessed during the 12-month baseline period prior to the index cardiac procedure. Distributions of all assessed covariates are provided in Additional file 1: Table S3

### Incidence of bleeding events

**Fig. 1:**
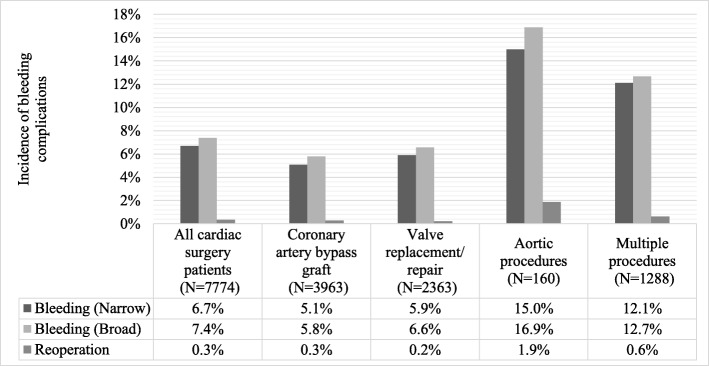
Incidence proportion of in-hospital bleeding complications (narrowly and broadly defined) and bleeding-related reoperation in cardiac surgery patients overall and by type of surgery

The incidence of in-hospital bleeding complications (narrow definition) was 6.7%. The bleeding incidence rate varied by type of cardiac surgery. Aortic procedures were associated with the highest bleeding complication rate (15.0%) followed by multiple cardiac procedures (12.1%), valve replacement/repair (5.9%), and CABG (5.1%). Figure 1 shows the incidence of bleeding complications (narrowly and broadly defined) and bleeding-related reoperation overall and by subgroup; the incidence of bleeding complication was slightly higher using the broader definition of bleeding complications. Bleeding-related reoperations were coded for only 0.3% of patients overall; 34.9% of reoperations occurred within the same episode of the cardiovascular surgery, whereas 65.1% occurred after the conclusion of the cardiovascular surgery episode; this number was substantially higher among patients who underwent aortic procedures (1.9%) compared to patients who underwent other types of cardiac surgery.

Additional file 1: Table S4 presents the results of the multivariable analyses examining the association between measured baseline characteristics and the risk of the primary (narrow) definition of bleeding complications. The following variables had a statistically significant association with bleeding complications: cardiac procedure type, more recent admission year, admission type, baseline hospitalization, baseline peripheral vascular disease, baseline renal disease, baseline diabetes, and baseline liver disease.

### Impact of bleeding complications on hospital LOS and critical care utilization

**Fig. 2:**
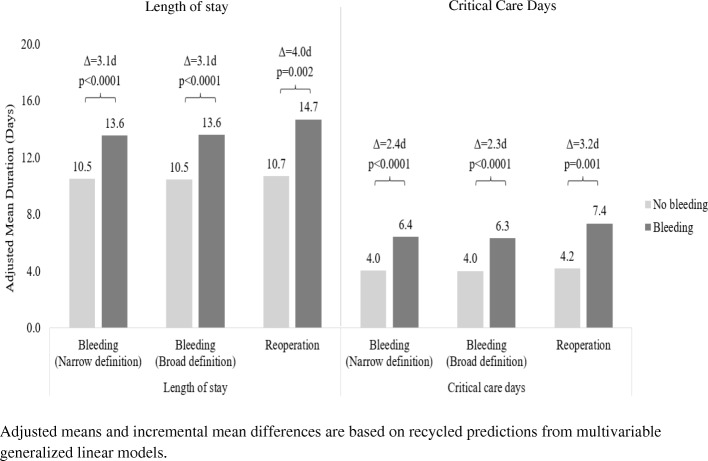
Adjusted mean length of stay and days in critical care by presence/absence of bleeding complications

The unadjusted mean post-procedure LOS was 15.4 days (SD = 14.4) and 10.4 days (SD = 8.9) among patients with and without bleeding complications, respectively. After adjustment for patient and procedure characteristics, the adjusted means were 13.6 days and 10.5 days for patients with and without bleeding complications, respectively (adjusted ratio = 1.29; 95% CI: 1.23, 1.36; *p* < 0.0001; MID = 3.1 days). Results were similar using the broad definition of bleeding complications (Fig. 2). Reoperation for bleeding complications was associated with a larger increase in mean post-procedure LOS: patients with a bleeding-relation reoperation had an adjusted mean LOS of 14.7 days versus 10.7 days in patients without a reoperation (adjusted ratio = 1.37; 95% CI: 1.12, 1.69; *p* = 0.002; MID = 4.0 days).

Eighty-seven percent of patients with bleeding complications spent one or more days in critical care following surgery (mean = 7.5 days, SD = 10.8), as compared with 82% of patients without bleeding complications (mean = 4.0 days, SD = 5.4). After adjustment for patient and procedure characteristics, the mean duration in critical care was 6.4 days and 4.0 days for patients with and without bleeding complications, respectively (adjusted ratio = 1.60; 95% CI: 1.47, 1.73; *p* < 0.0001; MID = 2.4 days). Results were similar using the broad definition of bleeding complications (Fig. 2). Reoperation for bleeding complications was associated with a larger increase in critical care days: patients with a bleeding-relation reoperation had an adjusted mean critical care duration of 7.4 days versus 4.2 days in patents without a reoperation (adjusted ratio = 1.76; 95% CI: 1.25, 2.46; *p* = 0.001; MID = 3.2 days).

Additional file 1: Tables S5 and S6 present the full results of the multivariable analyses examining the impact of the primary (narrow) definition of bleeding complications on hospital LOS and critical care utilization, respectively.

Aside from bleeding complications, the following variables had a statistically significant association with hospital LOS: sex, age, cardiac procedure type, cardiopulmonary bypass, admission type, baseline use of oral anticoagulants, baseline use of SSRIs, baseline use of SNRIs, baseline count of distinct prescription medications taken, baseline congestive heart failure, baseline peripheral vascular disease, baseline cerebrovascular disease, baseline chronic pulmonary disease, baseline peptic ulcer disease, baseline renal disease, baseline diabetes, baseline cancer, and baseline liver disease.

The following variables had a statistically significant association with critical care utilization: age, cardiac procedure type, cardiopulmonary bypass, admission type, baseline use of anticoagulants, baseline use of SSRIs, baseline count of distinct prescription medications taken, baseline anemia, baseline congestive heart failure, baseline peripheral vascular disease, baseline cerebrovascular disease, baseline chronic pulmonary disease, baseline renal disease, and baseline liver disease.

### Subgroup analyses

**Fig. 3:**
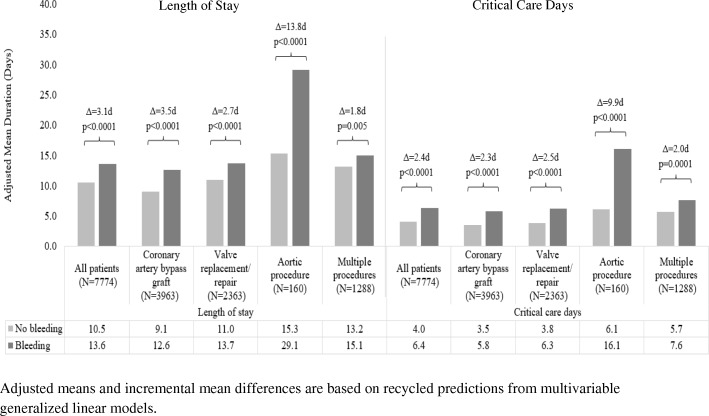
Adjusted mean length of stay and days in critical care by presence/absence of bleeding complications (narrow definition) and procedure type

In subgroup analyses, the impact of bleeding complications (narrowly defined) on LOS and critical care utilization in patients who underwent CABG, valve replacement/repair, and multiple procedures was similar in magnitude to what was observed in all cardiac surgery patients taken together (Fig. 3). Bleeding complications were associated with much larger increases in LOS (13.8 days; *p* < 0.0001) and critical care duration (9.9 days; *p* < 0.0001) in patients who underwent aortic procedures. Results by subgroup were similar when the broad definition of bleeding complications was considered (Additional file 2: Figure S1). When the impact of bleeding-related reoperations on healthcare utilization was evaluated by subgroup, reoperation was associated with a mean of 7.1 days longer LOS (*p* = 0.04) and 7.0 additional days in critical care (*p* = 0.009) in patients who underwent valve replacement/repair, but there were no significant associations with LOS or critical care utilization in the other subgroups (Additional file 3: Figure S2). However, the small number of reoperations documented in the study sample meant that we had limited power to detect differences based on reoperation status.

### In-hospital mortality

In the overall patient cohort, 218 patients (2.8%) died during the index cardiac surgery hospitalization. After covariate adjustment, in-hospital mortality was 7.1% in patients who experienced bleeding complications (narrowly defined) versus 2.3% in patients who did not (odds ratio = 3.44; *p* < 0.0001). The association between bleeding complications and in-hospital mortality, expressed as an odds ratio, varied from 2.66 in patients who underwent valve replacement/repair to 3.87 in patients who underwent multiple procedures. in-hospital mortality rates by surgery type and bleeding status are shown in Additional file 4: Figure S3.

### Sensitivity analyses

In the sensitivity analyses restricted only to patients with elective admissions, findings were similar to those of the primary analysis: patients with bleeding complications had longer LOS (MID: 2.6 days; *p* < 0.001) and spent more days in critical care (MID: 2.1 days; *p* < 0.001). In the propensity score matched analyses, patients were well-balanced on the matching variables after matching (as indicated by all standardized differences having an absolute value < 0.10; Additional file 5: Figure S4). As compared with the primary analyses, the propensity score matched analyses showed slightly larger differences in LOS and critical care days between patients with versus those without bleeding complications: patients with bleeding complications had longer LOS (MID: 3.3 days; *p* < 0.001) and spent more days in critical care (MID: 2.7 days; *p* < 0.001).

## Discussion

In this study of a large nationwide cohort of cardiac surgery patients treated at English hospitals in 2010–2016, in-hospital bleeding complications occurred in 6.7% of patients (based on narrow definition) and were associated with significant increases in hospital LOS (MID: 3.1 days) and critical care utilization (MID: 2.4 days). Reoperation for bleeding complications was documented in fewer patients (0.3%) but was associated with longer increases in hospital and critical care utilization (MID: 4.0 days and 3.2 days, respectively). These data provide contemporary real-world evidence of the economic burden associated with bleeding complications in cardiac surgery in English hospitals.

Bleeding complications were most frequent in patients who underwent aortic procedures or multiple cardiac procedures during the index hospitalization. The impact of bleeding complications on LOS and critical care utilization was largest among patients who underwent aortic operations. Among patients who underwent CABG, valve repair/replacement, or multiple procedures, the differences in healthcare utilization associated with bleeding complications were similar to the pattern that was observed in cardiac surgery patients overall.

Our results are largely consistent with prior evaluations of the impact of bleeding complications on healthcare utilization following cardiac surgery. In a single-center study of 1118 patients who underwent cardiac surgery with cardiopulmonary bypass at a large German teaching hospital in 2006, 6.4% of patients experienced excessive postoperative haemorrhage, defined as ≥200 ml/hour in any 1 h or ≥ 2 ml/kg/hour for 2 consecutive hours in the first 6 h after surgery. Excessive postoperative haemorrhage was associated with prolonged mechanical ventilation following surgery, a higher probability of intensive care unit (ICU) stay length > 72 h, greater ICU workload as measured by the Therapeutic Intervention Scoring System (TISS)-28, and a mean incremental increase of €6251 in total hospitalization costs. [1] A second study based on records from a French nationwide hospital discharge database included 15,450 patients who underwent CABG and 17,957 patients who underwent valve replacement in 2008, and identified bleeding complications using administrative diagnosis and procedure codes. Bleeding complications were recorded for 15.2% of CABG patients and 19.2% of valve replacement patients, and were associated with longer LOS in both groups (adjusted MID: 1.6 days in CABG and 2.1 days in valve replacement). [17] Relative to our study, in the French study bleeding complications were both more common and associated with smaller increases in healthcare utilization; a reasonable explanation is that their bleeding complications definition included some less-severe bleeding cases.

In our study, patients who underwent reoperation for bleeding had greater increases in LOS and critical care utilization than did the broader set of cardiac surgery patients who experienced bleeding complications. Studies that have looked specifically at reoperation to arrest bleeding following cardiac surgery have also found larger increases in healthcare utilization. One US-based study included 8586 patients who underwent CABG in 1992–1995 at one of five cardiac surgery centers in Maine, New Hampshire, and Vermont. Reoperation for bleeding was performed in 3.6% of patients. Patients who underwent reoperation had a significantly longer LOS following surgery relative to those without reoperation (unadjusted MID: 5.9 days). [3] In another study, the costs associated with postoperative haemorrhage were evaluated in 122 matched pairs of patients—one who experienced bleeding, one who did not—who underwent CABG at a US teaching hospital in 1992–1996. Bleeding complications were associated with a median increase of $3866 (1998 US$; $7589 in 2017 US$) in hospital costs. When patients were stratified by the approach used to control bleeding, it was found that costs were substantially higher in patients in whom a reoperation was performed ($9912; $19,456 in 2017 US$) relative to those who were managed medically ($3316; $6509 in 2017 US$). [18]

### Limitations

An important limitation of our study was that the identification of bleeding complications and bleeding-related reoperations was based on the presence of administrative diagnosis and procedure codes in a patient’s record. In general, surgical complications are likely to be under-coded in the HES-APC data and other hospital administrative databases. [19, 20] The incidence of bleeding complications observed in our study (6.7%) lies within the range of severe bleeding incidence estimates (2–15%) reported by prior studies with prospective collection of data on bleeding complications. [1, 2, 21–23] However, the incidence of reoperation for bleeding in our study (0.3%) was lower than estimates from prior studies (1–9%) that relied on chart reviews or prospective data collection to identify reoperations. [2–4, 24]. One explanation for this discrepancy is that immediate reopenings for bleeding before the patient leaves the operating theatre may be considered part of the index operation and not coded as a re-exploration for bleeding. Due to undercoding of bleeding complications and reoperations, we would expect that our incidence figures underestimate the true frequency of these events, and our measures of association between bleeding and healthcare utilization may also be underestimates due to misclassification.

In evaluating the relationship between bleeding complications and LOS, in-hospital mortality represents a competing risk that may influence the association. In-hospital mortality was substantially higher in patients who experienced bleeding complications (adjusted percentage: 7.1%) than in patients who did not (2.3%). In interpreting our findings, it should be kept in mind that the observed associations between bleeding complications and healthcare utilization provide an incomplete picture of the clinical relevance of bleeding complications, since LOS and critical care days are outcome measures that treat all discharges as equivalent.

Although the CPRD and HES-APC databases are the largest research databases of their kind available for England and have been characterized as representative of the UK general population in terms of age, sex, and ethnicity, these databases do not include all patients and therefore may not necessarily be generalizable to the entire population. [25] We were also unable to measure certain other factors which may influence hospital LOS, such as hospital routines regarding time of discharge and surgeon preferences. [26]

As noted above, prior research has shown that blood product transfusions are not well captured in the HES-APC database, and therefore were unavailable for characterization of the incidence or severity of bleeding complications in this study. [13] Furthermore, the database used for this study also does not contain other desirable clinical information, including details on anticoagulant consumption, use of dual antiplatelet therapy, time of discontinuation of these medications prior to surgery, use of antifibrinolytics and synthetic coagulation factor, baseline hematocrit, volume of bleeding, and amount of packed red blood cells. Future research is needed to determine the role that these clinical factors may play in the higher LOS and critical care utilization among individuals with vs. without bleeding complications.

## Conclusions

In conclusion, this study quantifies a substantial healthcare burden associated with bleeding complications in the setting of cardiac surgery. Strategies for reducing the incidence and/or severity of bleeding complications in cardiac surgery include better integration of peri-operative and point-of-care diagnostics and risk-stratification tools into the management of hemostatic function, [27–29] as well as appropriate use of pharmacologic antifibrinolytics, transfusion of hemostatic components, [27] and topical hemostatic agents [30, 31] to prevent and control bleeding. Future research is needed to evaluate the uptake and effectiveness of evidence-based interventions to reduce the incidence and/or severity of bleeding in cardiac surgery.

## Additional files

Additional file 1: **Table S1.** OPCS Classification of Surgical Operations and Procedures (4th revision) codes for identifying and classifying cardiac procedures. **Table S2.** Codes for bleeding complications and reoperation to arrest bleeding. **Table S3.** All baseline characteristics included as covariates included in multivariable regression models. **Table S4.** Multivariable analysis: association of baseline patient characteristics with bleeding complications (primary [narrow] definition). **Table S5.** Multivariable analysis: association of bleeding complications (primary [narrow] definition) with length of stay. **Table S6.** Multivariable analysis: association of bleeding complications (primary [narrow] definition) with critical care days. (DOCX 63 kb)

Additional file 2: **Figure S1.** Adjusted mean length of stay and days in critical care by presence/absence of bleeding complications (broad definition) and procedure type. (DOCX 62 kb)

Additional file 3: **Figure S2** Adjusted mean length of stay and days in critical care by presence/absence of reoperation for bleeding and procedure type. (DOCX 69 kb)

Additional file 4: **Figure S3.** In-hospital mortality rates by presence/absence of bleeding (narrow definition) and procedure type. (DOCX 57 kb)

Additional file 5: **Figure S4.** Standardized differences* before vs. after propensity score matching. *Each marker represents the standardized difference corresponding to a matching covariate used in the propensity score match. Black circles represent standardized differences before matching. Black triangles represent standardized differences after matching. Standardized differences with an absolute value < 0.10 are indicative of balance between matched groups. (DOCX 17 kb)

## Abbreviations

CABG: Coronary artery bypass graft
CPRD: Clinical Practice Research Datalink
EHR: Electronic health record
GP: General practitioner
HES-APC: Hospital Episode Statistics Admitted Patient Care
LOS: Length of stay (in this study, LOS refers specifically to post-procedure LOS)
MHRA: Medicines and Healthcare products Regulatory Agency
MID: Mean incremental difference
NHS: National Health Service
NSAID: Non-steroidal anti-inflammatory drug
SD: Standard deviation
SNRI: Serotonin-norepinephrine re-uptake inhibitor
SSRI: Selective serotonin re-uptake inhibitor
UK: United Kingdom
