# Supplementary material for: Impact of bleeding complications on length of stay and critical care utilization in cardiac surgery patients in England
Source: J Cardiothorac Surg. 2019 Apr 2;14:64. doi: 10.1186/s13019-019-0881-3 (PMC6444533; doi:10.1186/s13019-019-0881-3)
Supplement: Supplementary file 1 — Table S1. OPCS Classification of Surgical Operations and Procedures (4th revision) codes for identifying and classifying cardiac procedures. Table S2. Codes for bleeding complications and reoperation to arrest bleeding. Table S3. All baseline characteristics included as covariates included in multivariable regression models. Table S4. Multivariable analysis: association of baseline patient characteristics with bleeding complications (primary [narrow] definition). Table S5. Multivariable analysis: association of bleeding complications (primary [narrow] definition) with length of stay. Table S6. Multivariable analysis: association of bleeding complications (primary [narrow] definition) with critical care days. (DOCX 63 kb) [file 13019_2019_881_MOESM1_ESM.docx]

| **Appendix Table A1: OPCS Classification of Surgical Operations and Procedures (4th revision) codes for identifying and classifying cardiac procedures.** | | |
| --- | --- | --- |
| **Code** | **Description** | **Grouping** |
| K251 | Allograft replacement of mitral valve | Valve replacement/repair |
| K252 | Xenograft replacement of mitral valve | Valve replacement/repair |
| K253 | Prosthetic replacement of mitral valve | Valve replacement/repair |
| K254 | Replacement of mitral valve NEC | Valve replacement/repair |
| K255 | Mitral valve repair NEC | Valve replacement/repair |
| K258 | Other specified plastic repair of mitral valve | Valve replacement/repair |
| K259 | Unspecified plastic repair of mitral valve | Valve replacement/repair |
| K261 | Allograft replacement of aortic valve | Valve replacement/repair |
| K262 | Xenograft replacement of aortic valve | Valve replacement/repair |
| K263 | Prosthetic replacement of aortic valve | Valve replacement/repair |
| K264 | Replacement of aortic valve NEC | Valve replacement/repair |
| K265 | Aortic valve repair NEC | Valve replacement/repair |
| K268 | Other specified plastic repair of aortic valve | Valve replacement/repair |
| K269 | Unspecified plastic repair of aortic valve | Valve replacement/repair |
| K271 | Allograft replacement of tricuspid valve | Valve replacement/repair |
| K272 | Xenograft replacement of tricuspid valve | Valve replacement/repair |
| K273 | Prosthetic replacement of tricuspid valve | Valve replacement/repair |
| K274 | Replacement of tricuspid valve NEC | Valve replacement/repair |
| K276 | Tricuspid valve repair NEC | Valve replacement/repair |
| K279 | Unspecified plastic repair of tricuspid valve | Valve replacement/repair |
| K301 | Revision of plastic repair of mitral valve | Valve replacement/repair |
| K302 | Revision of plastic repair of aortic valve | Valve replacement/repair |
| K303 | Revision of plastic repair of tricuspid valve | Valve replacement/repair |
| K334 | Aortic root replacement using mechanical prosthesis | Aortic procedure |
| K335 | Aortic root replacement NEC | Aortic procedure |
| K338 | Other specified operations on aortic root | Aortic procedure |
| K401 | Saphenous vein graft replacement of one coronary artery | Coronary artery bypass graft |
| K402 | Saphenous vein graft replacement of two coronary arteries | Coronary artery bypass graft |
| K403 | Saphenous vein graft replacement of three coronary arteries | Coronary artery bypass graft |
| K404 | Saphenous vein graft replacement of four or more coronary arteries | Coronary artery bypass graft |
| K409 | Unspecified saphenous vein graft replacement of coronary artery | Coronary artery bypass graft |
| K411 | Autograft replacement of one coronary artery NEC | Coronary artery bypass graft |
| K412 | Autograft replacement of two coronary arteries NEC | Coronary artery bypass graft |
| K413 | Autograft replacement of three coronary arteries NEC | Coronary artery bypass graft |
| K414 | Autograft replacement of four or more coronary arteries NEC | Coronary artery bypass graft |
| K419 | Unspecified other autograft replacement of coronary artery | Coronary artery bypass graft |
| K421 | Allograft replacement of one coronary artery | Coronary artery bypass graft |
| K422 | Allograft replacement of two coronary arteries | Coronary artery bypass graft |
| K429 | Unspecified allograft replacement of coronary artery | Coronary artery bypass graft |
| K431 | Prosthetic replacement of one coronary artery | Coronary artery bypass graft |
| K433 | Prosthetic replacement of three coronary arteries | Coronary artery bypass graft |
| K441 | Replacement of coronary arteries using multiple methods | Coronary artery bypass graft |
| K442 | Revision of replacement of coronary artery | Coronary artery bypass graft |
| K449 | Unspecified other replacement of coronary artery | Coronary artery bypass graft |
| K451 | Double anastomosis of mammary arteries to coronary arteries | Coronary artery bypass graft |
| K452 | Double anastomosis of thoracic arteries to coronary arteries NEC | Coronary artery bypass graft |
| K453 | Anastomosis of mammary artery to left anterior descending coronary artery | Coronary artery bypass graft |
| K454 | Anastomosis of mammary artery to coronary artery NEC | Coronary artery bypass graft |
| K455 | Anastomosis of thoracic artery to coronary artery NEC | Coronary artery bypass graft |
| K456 | Revision of connection of thoracic artery to coronary artery | Coronary artery bypass graft |
| K459 | Unspecified connection of thoracic artery to coronary artery | Coronary artery bypass graft |
| L181 | Emergency replacement of aneurysmal segment of ascending aorta by anastomosis of aorta to aorta | Aortic procedure |
| L191 | Replacement of aneurysmal segment of ascending aorta by anastomosis of aorta to aorta NEC | Aortic procedure |
| L192 | Replacement of aneurysmal segment of thoracic aorta by anastomosis of aorta to aorta NEC | Aortic procedure |

| **Appendix Table A2: Codes for bleeding complications and reoperation to arrest bleeding.** | | | | |
| --- | --- | --- | --- | --- |
| **Concept** | **Code type** | **Code** | **Description** | **Notes** |
| Bleeding (narrow definition) | ICD-10 Dx | T810 | Haemorrhage and haematoma complicating a procedure, not elsewhere classified |  |
|  | OPCS | T032 | Reopening of chest and re-exploration of intrathoracic operation site and surgical arrest of postoperative bleeding |  |
|  | OPCS | Y321 | Re-exploration of organ and surgical arrest of postoperative bleeding NOC |  |
| Bleeding (broad definition) | ICD-10 Dx | T810 | Haemorrhage and haematoma complicating a procedure, not elsewhere classified |  |
|  | ICD-10 Dx | T812 | Accidental puncture and laceration during a procedure, not elsewhere classified |  |
|  | ICD-10 Dx | Y600 | Unintentional cut, puncture, perforation or haemorrhage during surgical and medical care--during surgical operation |  |
|  | ICD-10 Dx | Y605 | Unintentional cut, puncture, perforation or haemorrhage during surgical and medical care--during heart catheterization |  |
|  | ICD-10 Dx | Y608 | Unintentional cut, puncture, perforation or haemorrhage during surgical and medical care--during other surgical and medical care |  |
|  | ICD-10 Dx | Y609 | Unintentional cut, puncture, perforation or haemorrhage during surgical and medical care--during unspecified surgical and medical care |  |
|  | OPCS | T032 | Reopening of chest and re-exploration of intrathoracic operation site and surgical arrest of postoperative bleeding |  |
|  | OPCS | Y221 | Aspiration of haematoma of organ NOC |  |
|  | OPCS | Y321 | Re-exploration of organ and surgical arrest of postoperative bleeding NOC |  |
| Reoperation | OPCS | T032 | Reopening of chest and re-exploration of intrathoracic operation site and surgical arrest of postoperative bleeding |  |
|  | OPCS | T033 | Reopening of chest and re-exploration of intrathoracic operation site NEC | Requires T810 as concomitant diagnosis |
|  | OPCS | T034 | Reopening of chest NEC | Requires T810 as concomitant diagnosis |
|  | OPCS | Y221 | Aspiration of haematoma of organ NOC |  |
|  | OPCS | Y321 | Re-exploration of organ and surgical arrest of postoperative bleeding NOC |  |
|  | OPCS | Y322 | Re-exploration of organ and other repair of organ NOC | Requires T810 as concomitant diagnosis |
|  | OPCS | Y323 | Re-exploration of organ and packing of organ NOC | Requires T810 as concomitant diagnosis |
|  | OPCS | Y328 | Other specified re-exploration of organ NOC | Requires T810 as concomitant diagnosis |
|  | OPCS | Y329 | Unspecified re-exploration of organ NOC | Requires T810 as concomitant diagnosis |

| Appendix Table A3. All baseline characteristics included as covariates included in multivariable regression models. | | | |
| --- | --- | --- | --- |
| Covariate | All patients  (N=7,774) | No bleeding  (N=7,254) | Bleeding  (N=520) |
| Age in years |  |  |  |
| - 18-45 | 293 (4%) | 278 (4%) | 15 (3%) |
| - 46-64 | 2,187 (28%) | 2,063 (28%) | 124 (24%) |
| - 65 or older | 5,294 (68%) | 4,913 (68%) | 381 (73%) |
| Gender |  |  |  |
| - Male | 5,596 (72%) | 5,244 (72%) | 352 (68%) |
| - Female | 2,178 (28%) | 2,010 (28%) | 168 (32%) |
| Region   - North West | 179 (2%) | 165 (2%) | 14 (3%) |
| - North East | 1,302 (17%) | 1,189 (16%) | 113 (22%) |
| - Yorkshire & The Humber | 205 (3%) | 193 (3%) | 12 (2%) |
| - East Midlands | 74 (1%) | 68 (1%) | 6 (1%) |
| - West Midlands | 941 (12%) | 887 (12%) | 54 (10%) |
| - East of England | 849 (11%) | 759 (10%) | 90 (17%) |
| - South West | 1,082 (14%) | 1,014 (14%) | 68 (13%) |
| - South Central | 979 (13%) | 918 (13%) | 61 (12%) |
| - London | 982 (13%) | 933 (13%) | 49 (9%) |
| - South East Coast | 1,181 (15%) | 1,128 (16%) | 53 (10%) |
| Surgery type |  |  |  |
| - Coronary artery bypass graft (CABG) | 3,963 (51%) | 3,762 (52%) | 201 (38%) |
| - Valve replacement/repair | 2,363 (30%) | 2,224 (31%) | 139 (27%) |
| - Aortic procedure | 160 (2%) | 136 (2%) | 24 (5%) |
| - Multiple procedures† | 1,288 (17%) | 1,132 (16%) | 156 (30%) |
| Cardiopulmonary bypass |  |  |  |
| - Yes | 6,447 (83%) | 6,012 (83%) | 435 (84%) |
| - No | 1,327 (17%) | 1,242 (17%) | 85 (16%) |
| Admission type |  |  |  |
| - Elective | 5,400 (69%) | 5,099 (70%) | 301 (58%) |
| - Non-elective/emergency | 917 (12%) | 831 (11%) | 86 (17%) |
| - Transferred from another hospital | 1,457 (19%) | 1,324 (18%) | 133 (26%) |
| Year |  |  |  |
| - 2010 to 2011 | 2,978 (38%) | 2,762 (38%) | 216 (42%) |
| - 2012 to 2013 | 2,667 (34%) | 2,463 (34%) | 204 (39%) |
| - 2014 to February 2016 | 2,129 (27%) | 2,029 (28%) | 100 (19%) |
| Health conditions‡ | | | |
| - Myocardial infarction | 1,819 (23%) | 1,679 (23%) | 140 (27%) |
| - Congestive heart failure | 1,872 (24%) | 1,707 (24%) | 165 (32%) |
| - Cerebrovascular disease | 456 (6%) | 419 (6%) | 37 (7%) |
| - Peripheral vascular disease | 1,431 (18%) | 1,287 (18%) | 144 (28%) |
| - Diabetes | 1,951 (25%) | 1,842 (25%) | 109 (21%) |
| - Chronic pulmonary disease | 1,559 (20%) | 1,463 (20%) | 96 (18%) |
| - Renal disease | 1,003 (13%) | 902 (12%) | 101 (19%) |
| - Anemia | 287 (4%) | 265 (4%) | 22 (4%) |
| - Liver disease | 138 (2%) | 122 (2%) | 16 (3%) |
| - Peptic ulcer disease | 111 (1%) | 105 (1%) | 6 (1%) |
| - Connective tissue or rheumatic disease | 299 (4%) | 282 (4%) | 17 (3%) |
| - Cancer | 457 (6%) | 429 (6%) | 28 (5%) |
| - Dementia | 40 (1%) | 35 (0%) | 5 (1%) |
| - Paraplegia and hemiplegia | 26 (0%) | 24 (0%) | 2 (0%) |
| - HIV/AIDS | 1 (0%) | 1 (0%) | 0 (0%) |
| Medication use‡ |  |  |  |
| - Antiplatelet drugs | 4,681 (60%) | 4,382 (60%) | 299 (58%) |
| - Oral anticoagulant drugs | 1,001 (13%) | 936 (13%) | 65 (13%) |
| - Non-steroidal anti-inflammatory drugs (NSAIDs) | 448 (6%) | 428 (6%) | 20 (4%) |
| - Selective serotonin reuptake inhibitors (SSRIs) | 588 (8%) | 554 (8%) | 34 (7%) |
| - Serotonin-norepinephrine reuptake inhibitors (SNRIs) | 94 (1%) | 89 (1%) | 5 (1%) |
| Healthcare utilization‡ | | | |
| - Hospitalization | 2,968 (38%) | 2,725 (38%) | 243 (47%) |
| - ≥11 primary care encounters | 3,779 (49%) | 3,548 (49%) | 231 (44%) |
| - ≥6 distinct prescription medications | 2,319 (30%) | 2,157 (30%) | 162 (31%) |

† The multiple procedures category included 1,029 patients with CABG and valve replacement/repair and 259 patients who received other combinations, as detailed in the results section.

‡ Health conditions, medication use, and healthcare utilization were assessed during the 12-month baseline period prior to the index cardiac procedure.

**Appendix Table A4: Multivariable analysis: association of baseline patient characteristics with bleeding complications (primary [narrow] definition).**

|  | **Odds Ratio** | **95% Confidence Interval** | | **p-value** |
| --- | --- | --- | --- | --- |
| **Patient sex** |  | | | |
| Female | 1.22 | 0.99 | 1.50 | 0.057 |
| Male | reference category | | | |
| **Patient age category** |  | | | |
| 18-24 | 0.59 | 0.08 | 4.52 | 0.612 |
| 25-45 | 0.63 | 0.36 | 1.12 | 0.119 |
| 46-64 | 0.87 | 0.69 | 1.09 | 0.218 |
| ≥ 65 | reference category | | | |
| **Patient region of residence** |  | | | |
| East of England | 1.81 | 1.29 | 2.53 | 0.001 |
| London | 0.79 | 0.54 | 1.16 | 0.229 |
| Midlands | 0.92 | 0.64 | 1.32 | 0.646 |
| North | 1.40 | 1.03 | 1.91 | 0.033 |
| South Central | 0.93 | 0.64 | 1.34 | 0.688 |
| South East Coast | 0.71 | 0.49 | 1.03 | 0.073 |
| South West | reference category | | | |
| **Surgery type** |  | | | |
| Aortic aneurysm repair or aortic root replacement | 3.02 | 1.79 | 5.09 | <0.001 |
| Valve replacement/repair | 1.35 | 1.04 | 1.76 | 0.024 |
| Multiple Procedures | 2.67 | 2.08 | 3.42 | <0.001 |
| Coronary artery bypass graft | reference category | | | |
| **Cardiopulmonary bypass** | 0.92 | 0.71 | 1.18 | 0.507 |
| **Year of index hospitalization** |  | | | |
| 2011 | 1.02 | 0.77 | 1.35 | 0.912 |
| 2012 | 1.19 | 0.90 | 1.57 | 0.211 |
| 2013 | 0.88 | 0.65 | 1.18 | 0.391 |
| 2014 | 0.68 | 0.49 | 0.95 | 0.025 |
| 2015-2016 | 0.57 | 0.39 | 0.84 | 0.004 |
| 2010 | reference category | | | |
| **Admission type** |  |  |  |  |
| Non-Elective/ Emergency | 1.71 | 1.30 | 2.24 | <0.001 |
| Other Admission | 1.59 | 1.24 | 2.05 | <0.001 |
| Elective | reference category | | | |
| **Medication use** |  | | | |
| Oral anticoagulant drugs | 0.89 | 0.66 | 1.21 | 0.458 |
| Antiplatelet drugs | 0.99 | 0.81 | 1.22 | 0.940 |
| Selective serotonin reuptake inhibitors | 0.88 | 0.61 | 1.28 | 0.510 |
| Serotonin-norepinephrine reuptake inhibitors | 0.82 | 0.33 | 2.07 | 0.676 |
| Non-steroidal anti-inflammatory drugs | 0.71 | 0.45 | 1.14 | 0.156 |
| **Halthcare utilization** |  | | | |
| Baseline hospitalization | 1.23 | 1.00 | 1.50 | 0.049 |
| Count of face-to-face consultations | 1.00 | 0.99 | 1.01 | 0.811 |
| Count of distinct prescription medications | 1.01 | 0.97 | 1.04 | 0.615 |
| **Health conditions** |  | | | |
| Myocardial infarction | 1.17 | 0.92 | 1.49 | 0.210 |
| Congestive heart failure | 1.22 | 0.98 | 1.50 | 0.072 |
| Peripheral vascular disease | 1.28 | 1.02 | 1.61 | 0.034 |
| Cerebrovascular disease | 1.07 | 0.74 | 1.54 | 0.723 |
| Dementia | 1.78 | 0.67 | 4.76 | 0.248 |
| Chronic pulmonary disease | 0.81 | 0.64 | 1.03 | 0.083 |
| Connective tissue or rheumatic disease | 0.66 | 0.40 | 1.11 | 0.116 |
| Peptic ulcer disease | 0.64 | 0.27 | 1.50 | 0.304 |
| Renal disease | 1.49 | 1.16 | 1.91 | 0.002 |
| Diabetes | 0.73 | 0.58 | 0.92 | 0.009 |
| Cancer | 0.78 | 0.52 | 1.17 | 0.231 |
| Liver disease | 1.87 | 1.07 | 3.25 | 0.027 |
| Anemia | 0.98 | 0.61 | 1.57 | 0.933 |

**Appendix Table A5: Multivariable analysis: association of bleeding complications (primary [narrow] definition) with length of stay.**

|  | **Odds Ratio** | **95% Confidence Interval** | | **p-value** |
| --- | --- | --- | --- | --- |
| **Bleeding complication** | 1.29 | 1.23 | 1.36 | <0.001 |
| **Patient sex** |  | | | |
| Female | 1.05 | 1.02 | 1.08 | 0.002 |
| Male | reference category | | | |
| **Patient age category** |  | | | |
| 18-24 | 1.02 | 0.80 | 1.30 | 0.875 |
| 25-45 | 0.89 | 0.83 | 0.96 | 0.002 |
| 46-64 | 0.88 | 0.86 | 0.91 | <0.001 |
| ≥ 65 | reference category | | | |
| **Patient region of residence** |  | | | |
| East of England | 1.05 | 0.99 | 1.10 | 0.095 |
| London | 1.11 | 1.05 | 1.16 | <0.001 |
| Midlands | 1.02 | 0.97 | 1.07 | 0.364 |
| North | 1.01 | 0.96 | 1.05 | 0.758 |
| South Central | 1.07 | 1.02 | 1.13 | 0.005 |
| South East Coast | 1.05 | 1.01 | 1.11 | 0.028 |
| South West | reference category | | | |
| **Surgery type** |  | | | |
| Aortic aneurysm repair or aortic root replacement | 1.67 | 1.52 | 1.82 | <0.001 |
| Valve replacement/repair | 1.16 | 1.12 | 1.20 | <0.001 |
| Multiple Procedures | 1.30 | 1.25 | 1.35 | <0.001 |
| Coronary artery bypass graft | reference category | | | |
| **Cardiopulmonary bypass** | 1.13 | 1.09 | 1.16 | <0.001 |
| **Year of index hospitalization** |  | | | |
| 2011 | 1.02 | 0.98 | 1.06 | 0.359 |
| 2012 | 1.06 | 1.02 | 1.11 | 0.005 |
| 2013 | 1.03 | 0.98 | 1.07 | 0.209 |
| 2014 | 0.98 | 0.94 | 1.02 | 0.382 |
| 2015-2016 | 0.93 | 0.89 | 0.97 | 0.002 |
| 2010 | reference category | | | |
| **Admission type** |  |  |  |  |
| Non-Elective/ Emergency | 1.27 | 1.22 | 1.33 | <0.001 |
| Other Admission | 1.18 | 1.14 | 1.23 | <0.001 |
| Elective | reference category | | | |
| **Medication use** |  | | | |
| Oral anticoagulant drugs | 1.10 | 1.06 | 1.15 | <0.001 |
| Antiplatelet drugs | 0.99 | 0.96 | 1.02 | 0.458 |
| Selective serotonin reuptake inhibitors | 1.06 | 1.01 | 1.11 | 0.020 |
| Serotonin-norepinephrine reuptake inhibitors | 1.25 | 1.12 | 1.40 | <0.001 |
| Non-steroidal anti-inflammatory drugs | 0.99 | 0.94 | 1.05 | 0.808 |
| **Halthcare utilization** |  | | | |
| Baseline hospitalization | 1.00 | 0.97 | 1.03 | 0.870 |
| Count of face-to-face consultations | 1.00 | 1.00 | 1.00 | 0.721 |
| Count of distinct prescription medications | 1.01 | 1.01 | 1.02 | <0.001 |
| **Health conditions** |  | | | |
| Myocardial infarction | 1.01 | 0.97 | 1.04 | 0.632 |
| Congestive heart failure | 1.19 | 1.16 | 1.23 | <0.001 |
| Peripheral vascular disease | 1.10 | 1.07 | 1.14 | <0.001 |
| Cerebrovascular disease | 1.13 | 1.07 | 1.20 | <0.001 |
| Dementia | 1.03 | 0.86 | 1.22 | 0.773 |
| Chronic pulmonary disease | 1.07 | 1.04 | 1.10 | <0.001 |
| Connective tissue or rheumatic disease | 0.95 | 0.89 | 1.01 | 0.120 |
| Peptic ulcer disease | 1.29 | 1.16 | 1.42 | <0.001 |
| Renal disease | 1.19 | 1.14 | 1.23 | <0.001 |
| Diabetes | 1.05 | 1.02 | 1.09 | 0.001 |
| Cancer | 1.07 | 1.01 | 1.13 | 0.018 |
| Liver disease | 1.20 | 1.09 | 1.32 | <0.001 |
| Anemia | 0.99 | 0.93 | 1.06 | 0.779 |

**Appendix Table A6: Multivariable analysis: association of bleeding complications (primary [narrow] definition) with critical care days.**

|  | **Odds Ratio** | **95% Confidence Interval** | | **p-value** |
| --- | --- | --- | --- | --- |
| **Bleeding complication** | 1.59 | 1.47 | 1.73 | <0.001 |
| **Patient sex** |  | | | |
| Female | 0.99 | 0.94 | 1.04 | 0.5913 |
| Male | reference category | | | |
| **Patient age category** |  | | | |
| 18-24 | 0.83 | 0.54 | 1.26 | 0.3834 |
| 25-45 | 0.81 | 0.72 | 0.92 | 0.0010 |
| 46-64 | 0.94 | 0.90 | 0.99 | 0.0238 |
| ≥ 65 | reference category | | | |
| **Patient region of residence** |  | | | |
| East of England | 0.99 | 0.91 | 1.08 | 0.8796 |
| London | 1.10 | 1.02 | 1.20 | 0.0199 |
| Midlands | 1.07 | 0.98 | 1.16 | 0.1171 |
| North | 0.80 | 0.74 | 0.86 | <0.001 |
| South Central | 1.15 | 1.06 | 1.25 | 0.0008 |
| South East Coast | 0.95 | 0.88 | 1.03 | 0.2507 |
| South West | reference category | | | |
| **Surgery type** |  | | | |
| Aortic aneurysm repair or aortic root replacement | 1.77 | 1.52 | 2.06 | <0.001 |
| Valve replacement/repair | 1.06 | 1.00 | 1.13 | 0.0367 |
| Multiple Procedures | 1.42 | 1.33 | 1.51 | <0.001 |
| Coronary artery bypass graft | reference category | | | |
| **Cardiopulmonary bypass** | 1.21 | 1.14 | 1.28 | <0.001 |
| **Year of index hospitalization** |  | | | |
| 2011 | 1.20 | 1.12 | 1.28 | 1.20 |
| 2012 | 1.17 | 1.09 | 1.25 | 1.17 |
| 2013 | 1.08 | 1.00 | 1.16 | 1.08 |
| 2014 | 0.99 | 0.91 | 1.06 | 0.99 |
| 2015-2016 | 1.07 | 0.99 | 1.16 | 1.07 |
| 2010 | reference category | | | |
| **Admission type** |  |  |  |  |
| Non-Elective/ Emergency | 1.28 | 1.20 | 1.37 | <0.001 |
| Other Admission | 1.24 | 1.17 | 1.32 | <0.001 |
| Elective | reference category | | | |
| **Medication use** |  | | | |
| Oral anticoagulant drugs | 1.09 | 1.01 | 1.16 | 0.0204 |
| Antiplatelet drugs | 1.02 | 0.97 | 1.07 | 0.5587 |
| Selective serotonin reuptake inhibitors | 1.13 | 1.04 | 1.23 | 0.0025 |
| Serotonin-norepinephrine reuptake inhibitors | 1.18 | 0.97 | 1.42 | 0.0943 |
| Non-steroidal anti-inflammatory drugs | 0.91 | 0.83 | 1.00 | 0.0590 |
| **Halthcare utilization** |  | | | |
| Baseline hospitalization | 0.97 | 0.92 | 1.02 | 0.1772 |
| Count of face-to-face consultations | 1.00 | 1.00 | 1.00 | 0.2624 |
| Count of distinct prescription medications | 1.01 | 1.00 | 1.02 | 0.0063 |
| **Health conditions** |  | | | |
| Myocardial infarction | 1.00 | 0.95 | 1.07 | 0.8807 |
| Congestive heart failure | 1.23 | 1.17 | 1.30 | <0.001 |
| Peripheral vascular disease | 1.12 | 1.06 | 1.18 | 0.0002 |
| Cerebrovascular disease | 1.17 | 1.07 | 1.28 | 0.0008 |
| Dementia | 0.98 | 0.73 | 1.30 | 0.8843 |
| Chronic pulmonary disease | 1.11 | 1.05 | 1.17 | <0.001 |
| Connective tissue or rheumatic disease | 0.98 | 0.88 | 1.09 | 0.6953 |
| Peptic ulcer disease | 1.18 | 0.99 | 1.41 | 0.0588 |
| Renal disease | 1.36 | 1.28 | 1.45 | <0.001 |
| Diabetes | 1.03 | 0.98 | 1.09 | 0.2448 |
| Cancer | 1.05 | 0.96 | 1.15 | 0.2494 |
| Liver disease | 1.58 | 1.36 | 1.84 | <0.001 |
| Anemia | 0.84 | 0.75 | 0.95 | 0.0036 |
