# Supplementary figures and images for: Impact of bleeding complications on length of stay and critical care utilization in cardiac surgery patients in England
Source: J Cardiothorac Surg. 2019 Apr 2;14:64. doi: 10.1186/s13019-019-0881-3 (PMC6444533; doi:10.1186/s13019-019-0881-3)

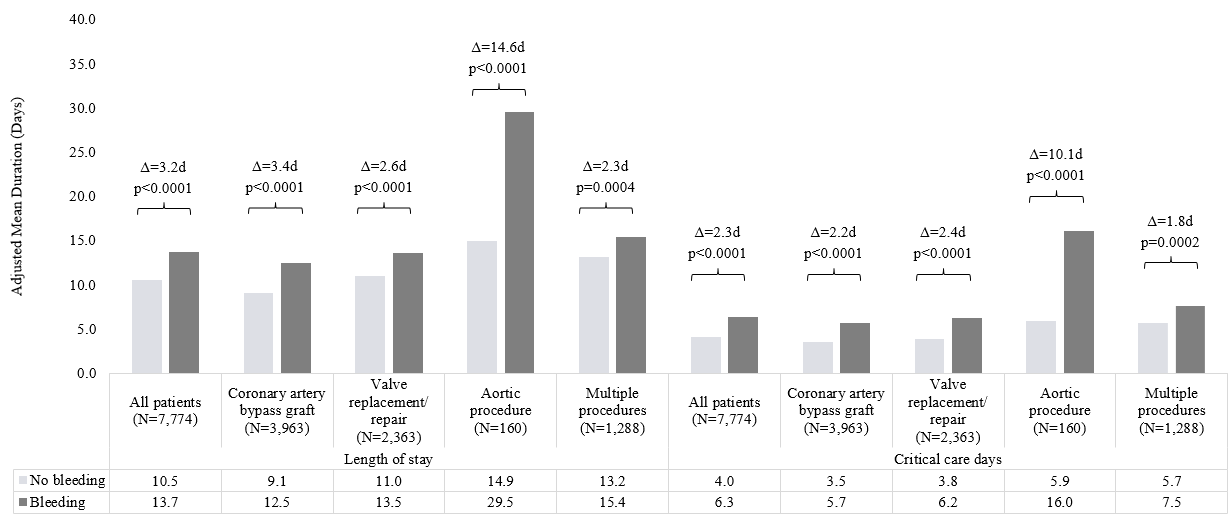


Critical Care Days

Length of Stay

Supplement: Supplementary file 2 — Figure S1. Adjusted mean length of stay and days in critical care by presence/absence of bleeding complications (broad definition) and procedure type. (DOCX 62 kb) [file 13019_2019_881_MOESM2_ESM.docx]

Length of Stay


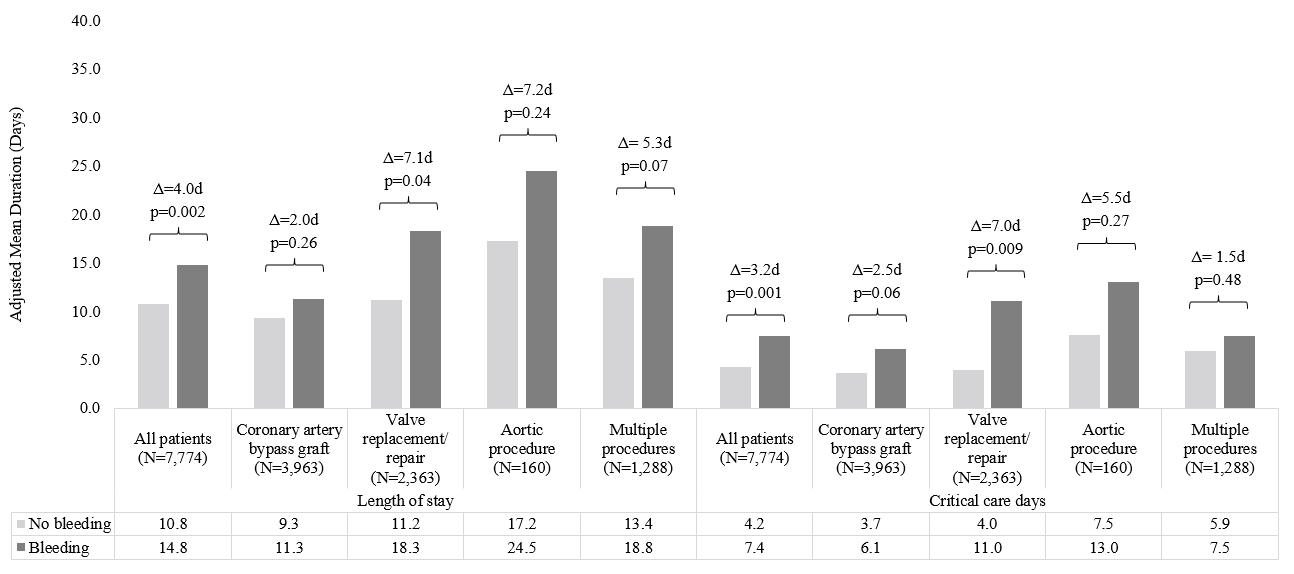


Critical Care Days

Supplement: Supplementary file 3 — Figure S2 Adjusted mean length of stay and days in critical care by presence/absence of reoperation for bleeding and procedure type. (DOCX 69 kb) [file 13019_2019_881_MOESM3_ESM.docx]
