# Supplementary material for: Impact of bleeding complications on length of stay and critical care utilization in cardiac surgery patients in England
Source: J Cardiothorac Surg. 2019 Apr 2;14:64. doi: 10.1186/s13019-019-0881-3 (PMC6444533; doi:10.1186/s13019-019-0881-3)
Supplement: Supplementary file 4 — Figure S3. In-hospital mortality rates by presence/absence of bleeding (narrow definition) and procedure type. (DOCX 57 kb) [file 13019_2019_881_MOESM4_ESM.docx]

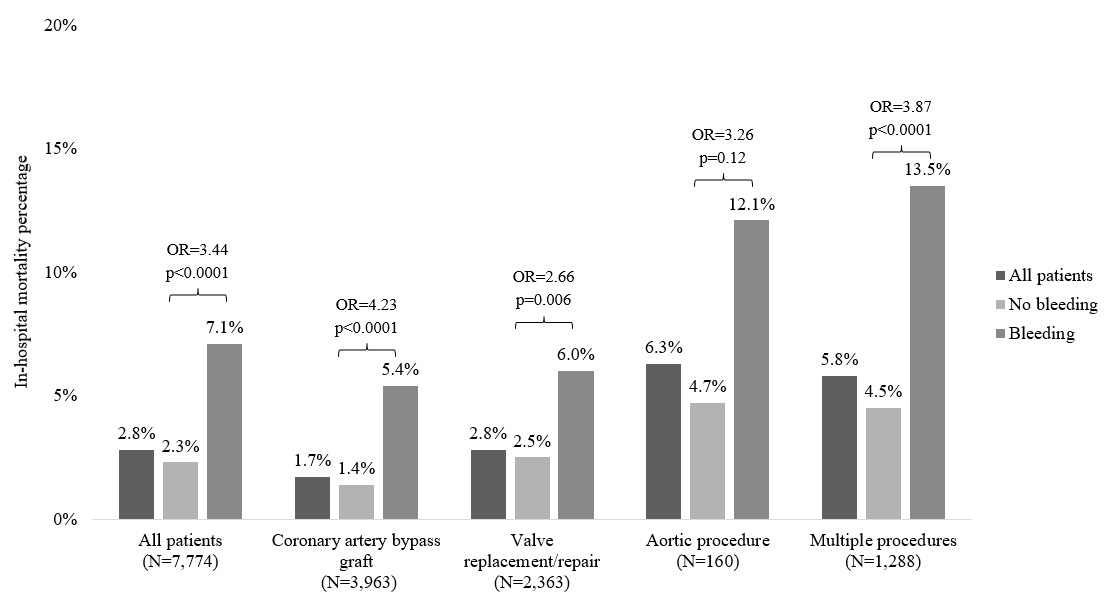


Percentages reflect unadjusted mortality rates (for all patients unstratified by bleeding status) and multivariable-adjusted mortaltiy rates for patients with and without in-hospital bleeding (narrowly defined). Multivariable adjusted odds ratios (ORs) and p-values reflecting the difference in mortality by bleeding status are provided for the entire cohort and for individual procedure types.
