# Supplementary material for: Impact of bleeding complications on length of stay and critical care utilization in cardiac surgery patients in England
Source: J Cardiothorac Surg. 2019 Apr 2;14:64. doi: 10.1186/s13019-019-0881-3 (PMC6444533; doi:10.1186/s13019-019-0881-3)
Supplement: Supplementary file 5 — Figure S4. Standardized differences* before vs. after propensity score matching. *Each marker represents the standardized difference corresponding to a matching covariate used in the propensity score match. Black circles represent standardized differences before matching. Black triangles represent standardized differences after matching. Standardized differences with an absolute value < 0.10 are indicative of balance between matched groups. (DOCX 17 kb) [file 13019_2019_881_MOESM5_ESM.docx]

*Each marker represents the standardized difference corresponding to a matching covariate used in the propensity score match. Black circles represent standardized differences before matching. Black triangles represent standardized differences after matching. Standardized differences with an absolute value <0.10 are indicative of balance between matched groups.
